# Recent progress in the role of autophagy in neurological diseases

**DOI:** 10.15698/cst2019.05.186

**Published:** 2019-04-29

**Authors:** Tian Meng, Shiyin Lin, Haixia Zhuang, Haofeng Huang, Zhengjie He, Yongquan Hu, Qing Gong, Du Feng

**Affiliations:** 1 State Key Laboratory of Respiratory Disease, School of Basic Medical Sciences, Guangzhou Medical University; Affiliated Cancer Hospital of Guangzhou Medical University, Guangzhou 511436, China.; 2 Institute of Neurology, Guangdong Key Laboratory of Age-Related Cardiac-Cerebral Vascular Disease, Affiliated Hospital of Guangdong Medical College, Zhanjiang, Guangdong, China.; 3 Department of Biochemistry and Molecular Biology, GMU-GIBH Joint School of Life Sciences, Guangzhou Medical University, Guangzhou 511436, People's Republic of China.

**Keywords:** autophagy, neuro-degenerative diseases, mTOR, Parkinson's disease, Alzheimer's disease, Huntington's disease, Amyotrophic lateral sclerosis

## Abstract

Autophagy (here refers to macroautophagy) is a catabolic pathway by which large protein aggregates and damaged organelles are first sequestered into a double-membraned structure called autophago-some and then delivered to lysosome for destruction. Recently, tremen-dous progress has been made to elucidate the molecular mechanism and functions of this essential cellular metabolic process. In addition to being either a rubbish clearing system or a cellular surviving program in response to different stresses, autophagy plays important roles in a large number of pathophysiological conditions, such as cancer, diabetes, and especially neurodegenerative disorders. Here we review recent progress in the role of autophagy in neurological diseases and discuss how dysregulation of autophagy initiation, autophagosome formation, maturation, and/or au-tophagosome-lysosomal fusion step contributes to the pathogenesis of these disorders in the nervous system.

## AUTOPHAGY

Autophagy is an evolutionary conserved cellular process, which is characterized by the appearance of double-membrane autophagosomes sequestering portions of cellular organelles and cytoplasm and subsequently delivering them to the lysosome for degradation [1, 2]. After destruction of the autophagic cargo, amino acids, nucleotides, fatty acids, sugars, the building blocks are released into the cytosol and reutilized in metabolic pathways [3]. Therefore, autophagy is crucial for maintaining cellular homeostasis as well as remodeling during normal development, and plays a critical role in overcoming adverse conditions, such as starvation and intrinsic or extrinsic cellular stresses (hypoxia, reactive oxygen species accumulation, endoplasmic reticulum stress and bacterial infections) [4]. Dysfunctions in autophagy have been associated with a variety of pathologies including cancer [5–8], neurodegenerative diseases [9–13], inflammatory diseases [14, 15], metabolic diseases [6, 16, 17] and heart dysfunction [18–20].

**Figure 1:**
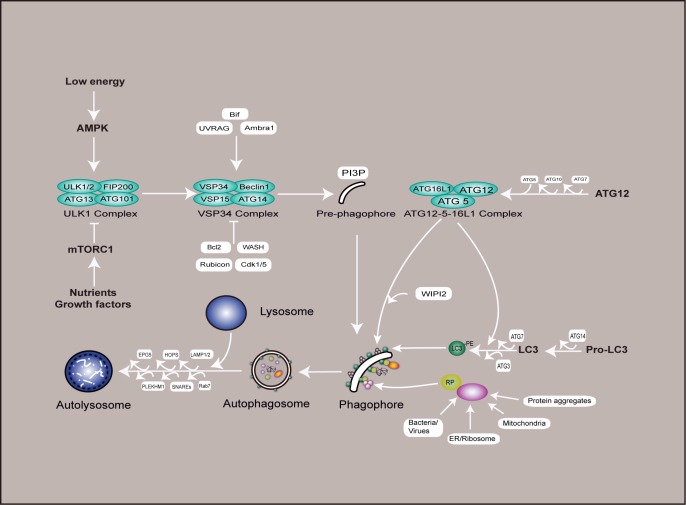
FIGURE 1: Schematic of the mammalian autophagy pathway. This diagram shows a simplified version of autophagy. Nutrient or growth factor deprivation and low energy are well established autophagy inducers, leading to AMPK activation and mTORC1 inhibition, which positively trigger the formation of ULK1 complex (ULK1, ULK2, ATG13, FIP200 and ATG101). This complex subsequently activates the VSP34 complex (VSP34, Beclin1, VSP15 and ATG14) to promote PI3P synthesis in pre-autophagosomal structures, thus the initiation of autophagy has been activated. PI3P specifically binds its effector WIPI2 and catalyzes two types of ubiquitination-like reactions that are in charge of the extension and closure of the autophagosome double membranes. In the first reaction, ATG12 and ATG5 are conjugated to each other in the presence of ATG7 and ATG10, and ATG16L subsequently binds to them to form the ATG12–ATG5–ATG16L1 complex. In the second reaction, LC3-I and PE are conjugated to membranes in the presence of ATG14, ATG7 and ATG3, this process is facilitated by the ATG12–ATG5–ATG16L1 complex, ultimately leading to the formation of the complete autophagosome. Receptor proteins such as p62, NDP52, and NBR1 are responsible for the recognition of cytoplasmic targets (e.g., protein aggregates, damaged mitochondria, ER/ribosome, and infectious agents), and establish a bridge between LC3-II and specific ubiquitinated cargos to sustain the engulfment of a variety of substrates. In the final step of the process, the completed autophagosomes are then trafficked to fuse with lysosomes, resulting in the degradation of the vesicle contents, and this process is regulated by LAMP1/2, EPG5, HOPS, PLEKHM1 and SNAREs. AMPK - AMP-activated protein kinase; mTORC1 - mechanistic target of rapamycin complex 1; ULK1 - Unc-51-like kinase; ATG - autophagy protein; VPS34 - phosphatidylinositol 3-kinase VPS34; PI3P - phosphatidylinositol 3-phosphate; PE - phosphatidylethanolamine; RP - receptor protein.

The formation of the autophagosome is dominated by a series of autophagy-related (ATG) genes and protein complexes acting sequentially, so that autophagy is induced when needed, but otherwise maintained at a basal level. The ULK1 complex (ULK1/2–ATG13–FIP200–ATG101) is in charge of autophagy induction, the class III phosphatidylinositol 3-kinase (PI3K)/VPS34 complex (VPS34, Beclin 1, ATG14 and VSP15 form the core of this complex, while Bif, Ambra1 and UVRAG, positively regulate its activity) is in charge of autophagosome initiation, ATG12–ATG5–ATG16L1 and LC3-I/LC3-PE (LC3-II) complexes are in charge of the extension and closure of the autophagosome double membranes (**Figure 1**). After autophagosome maturation, its outer membrane fuses with the lysosome membrane, the inner membrane and contents are degraded by hydrolases in the lysosome, thus generating amino acids and other cellular building blocks recycled by the cell, and this process is also a quality control mechanism for cellular organelles and proteins [21–23].

## THE REGULATION OF AUTOPHAGY

Besides the core components mentioned above, autophagy is regulated by important nutrient-sensing pathways including the mammalian target of rapamycin complex 1 (mTORC1) and AMP-activated protein kinase (AMPK), which oppositely modulate the ULK1 complex via a series of phosphorylation events, inhibiting or activating autophagy, respectively (**Figure 1**).

Nutrients and growth factor (signaled by receptor tyrosine kinases and PI3K/AKT pathway) availability are sensed by mTORC1, which inhibits autophagy by phosphorylating both ATG13 and ULK1 at Ser 757 and by binding to the ULK1 complex, thereby inhibiting the activity of ULK1 kinase and blocking the formation of the phagophore [24–28]. On the other hands, low cellular energy levels activate AMPK by phosphorylation of Thr 172 and subsequently stimulates autophagy through activating ULK1 by phosphorylation of Ser 77 and Ser 317 under glucose deprivation [29] or Ser 555 under mitophagy and amino acid starvation [30]. Activation of the ULK1 complex leads to the recruitment of VPS34 to the phagophore initiation sites, thus stimulating the formation of the phosphatidylinositol 3-phosphate (PI3P) complex and the production of PI3P, which, in turn, helps to recruit ATG16L1 by WIPI2 to autophagosome formation sites [31]. Some signals act via the ATG6 orthologous Beclin 1, which promotes VPS34 activity [32, 33]. While Bcl2, WASH, Rubicon, and Cdk1/5 negatively regulate the PI3P complex to suppress autophagy [34, 35].

The ATG12–ATG5–ATG16L1 complexes and ATG8/LC3 ubiquitin-like conjugation systems are required for maintaining the phagophore expansion. Prior to this process, ATG12 is activated by ATG7 (E1-like enzyme), attached to ATG5 by ATG10 (E2-like enzyme) and then to ATG16L, and finally enters the phagophore as a ATG5-ATG12-ATG16L1 complex. LC3 also undertakes an analogous processing: it is first cleaved by ATG4, which exposes a glycine residue by cleaving the C terminus of LC3 (LC3-I), and then conjugated to the lipid phosphatidylethanolamine (PE) with the help of ATG7, ATG3 and the ATG5-ATG12-ATG16L1 complex, leading to LC3-II formation. This process is closely associated with the extension and closure of the autophagosome double membranes [36]. The recognition of cytoplasmic targets is aided by receptor proteins, such as p62/SQSTM1 (ATG19 in yeast) [37, 38], OPTN [39], NDP52 [40], NBR1 [41], ALFY [42], and TRIM5 [43], which bind to ubiquitinated proteins and link them to LC3 in the phagophore. Fusion of autophagosomes with lysosomes is supported by Rab7 GTPase and the lysosomal associated membrane protein 1/2 (LAMP1/2) [44, 45], where EPG5 [46, 47], HOPS[48, 49], PLEKHM1[50]and SNAREs[51] are also required. Autophagolysosomal contents are decomposed by lysosomal acid hydrolases, including Cathepsin B, D, and L [52–54].

## AUTOPHAGY AND THE NERVOUS SYSTEM

The brain is often the most severely affected organ in most lysosome disorders and mutations in genes involved in autophagy pathways are usually linked to neurodegenerative disorders, indicating the heavy reliance of neurons on autophagy to maintain normal function and homeostasis.

In neurons, autophagic vacuoles (AVs) are generated in axons while lysosomes are concentrated mainly near the cell body, which means that there are long distances between AVs and lysosomes due to the large expanses of dendritic and axonal cytoplasm [55]. In addition, the dysfunction of cell division in neurons causes particular obstacles in preventing impaired organelles and other waste from accumulating over a life time. In contrast, mitotic cells can dilute these waste burdens by cell division [56, 57].Thus, neurons are particularly vulnerable for gradually losing the ability of efficiently clearing those burdens due to aging, which eventually results in abnormal accumulation of autolysosomal substrates like ubiquitinated protein aggregates, resulting in degeneration of neurons[30, 58].

Although those ubiquitinated substrates can be cleared through autophagy or the ubiquitin proteasome system, autophagy is the only route to degrade large impaired organelles or protein aggregates, because they are too large to go into the narrow entrance of the proteasome chamber in the proteasome pathway. This highlights the essential role of autophagy in protein degradation and recycling in the mammalian nervous system.

## AUTOPHAGY IN NEURODEGENERATIVE DISEASES

Increasing evidence has confirmed the importance of autophagy in neuronal health, and a strong link between autophagy and neurodegenerative diseases has been established based on its role of clearing abnormal aggregated proteins [59]. In fact, the intra neuronal aggregated proteins, which appear in the most late-onset neurodegenerative diseases, are usually the substrates for autophagic degradation [60, 61]. The vast majority of neurodegenerative diseases, including sporadic forms and familial forms, are associated with inherited genetic mutations, and the assessment of the functions of these disease-associated genes has indicated autophagic dysfunction in pathogenesis [61]. However, the contribution of autophagy dysfunction to neurodegenerative disease progression is unknown.

## ALZHEIMER'S DISEASE

Alzheimer's disease (AD) is the most common neurodegenerative disease that is characterized by extensive loss of cognitive functions. The main pathological hallmarks of AD are extracellular senile plaques which are composed of aggregated β-amyloid (Aβ) and intracellular neurofibrillary tangles (NFT) which are made of aggregated hyperphosphorylated tau protein [62].

Aβ originates from proteolysis of the amyloid precursor protein (APP) by the sequential enzymatic actions of β-site APP-cleaving enzyme 1 (BACE1), β-secretase, and γ-secretase, a protein complex with presenilin 1 (PS1) at its catalytic core. In AD brains, a high level of APP proteins, Aβ and PS1, accumulate in AVs in swollen dystrophic neurites, and autophagy activation was elevated after Aβ stimulation or in APP/PS1 mice, a mouse model of AD, indicating that autophagy is implicated in AD pathogenesis [63–65].There is a complex relation between Aβ and autophagy. Aβ may be generated in AVs during autophagy, ATG7 deletion results in lower Aβ extracellular secretion and plaque formation in APP transgenic mice [66], suggesting that the activation of autophagy may further exacerbate AD pathogenesis in AD brains [65]. However, Aβ may also be degraded by autophagy, it has been reported that enhancement of autophagy can reduce Aβ levels in a number of systems [67–70].

Hyperphosphorylated tau accumulation into intracellular tangles is another pathological hallmark of AD, and is also found in other neuronal diseases, such as frontotemporal dementias (FTDs) [71]. Abnormal tau disrupts vesicle transport in axons by destroying the dynein-dynactin complex, raising the number of autophagosomes and leading to tau-induced toxicity in AD and FTDs [72, 73]. New data suggested that autophagy is able to degrade both soluble and aggregated forms of tau. Thus, the inhibition of autophagy accelerates tau aggregation and toxicity, and in contrast, treatment with rapamycin, an autophagy activator, decreases tau pathology [74–76]. This could be further confirmed by the studies in transgenic mouse models, and the results indicated that autophagy activation can suppress the formation of tau pathology and subsequently ameliorate cognitive deficits [77–79]. Moreover, impaired lysosomal membrane integrity was also emerged in AD patients [80], and tau has been reported to perturb lysosomal permeability by binding the membrane of lysosomes both *in vitro* and *in vivo* [76, 81].

In healthy neurons, AVs are efficiently disposed, but in AD brains the impaired clearance of AVs, not induction of autophagy itself, results in the accumulation of autophagic vacuoles [30], indicating that the adjustment regulation of the late steps of autophagy could be a possible therapeutic strategy for AD. A further study demonstrated that the enhancement of AVs is found in PS1-rich locations [82], and knock down of PS1 leads to defects in autophagosome clearance, lysosomal acidification, and lysosomal proteolytic activity. Mutations of PS1 result in similar abnormalities in the autolysosomal pathway and are associated with early-onset AD [83]. Moreover, lack of phosphorylation on Ser367 of PS1 blocks the fusion of autophagosome and lysosome, and leads to Aβ accumulation in the mouse brain by reducing β C-terminal fraction (CTF) degradation [84]. A recent study points out that phosphorylated PS1 is capable of interacting with Annexin A2 which regulates the autophagosome-autolysosome fusion by promoting the combination of Vamp8 and autophagosomal SNARE Syntaxin 17 [84]. Based on these observations, it is reasonable to propose that recovering lysosome function may enhance the clearance of protein aggregates. And this can be further confirmed by the results that the deletion of cystatin B, an inhibitor of lysosome cysteine proteases, promotes the removal of aberrant protein aggregations in lysosomes of AD mice [85].

Recently, new mechanistic insights proposed that autophagic pathology in AD is caused by abnormal axonal retrograde transport of AVs. Aβ oligomers can bind to dynein intermediate chain (DIC) and cause the deficiency of dynein motors, which block its function of providing motility for retrograde transport and sending AVs to lysosomes for digestion. Hence, retrograde transport of axonal autophagosomes is obstructed and leads to autophagic stress in AD neurons [86].

Beclin1, the key factor in autophagosome formation, has been shown to be suppressed both on mRNA and protein levels in AD brains [87, 88]. A study showed that the decline of Beclin1 on protein level is caused by caspase 3 cleavage, which is activated in AD patients brains and leads to autophagy disruption [89]. Nrf2, a vital transcription factor for regulating autophagy related protein transcription [90], could stimulate autophagy by inducing autophagy receptor NDP52 and lower aggregated tau proteins in response to oxidative stress [91]. Based on these results, the levels of Beclin1 and Nrf2 are thought to be regarded as common potential markers for pathology of AD.

It has been identified by genetic studies that phosphatidylinositol binding clathrin assembly protein (PICALM) is involved in autophagy [92, 93], and changes in the level of this protein have been found in AD patients brains [94, 95]. PICALM is a clathrin adaptor protein, and is involved in endocytic trafficking by regulating endocytosis of soluble NSF attachment protein receptors (SNAREs), thus enhancing autophagy to clear tau aggregations [96]. In addition, PICALM could act as an autophagy receptor when compounded with assembly polypeptide 2 (AP2), that own the function of interacting with LC3 and targeting APP into autophagosomes [97].

Researchers have found that many proteins prevent or promote AD progression via autophagy pathway. Nuclear receptor binding factor 2 (NRBF2) is a component of PI3K complex and involve in the regulation of autophagy. A study discovered that NRBF2 is reduced in the hippocampus of 5XFAD mice. However, knockout of NRBF2 can increase the level and half-life of APP-CTFs, Aβ_1-40_ and Aβ_1-42_ apparently, which demonstrates that NRBF2 plays an important role in the degradation of APP-CTFs and Aβ. In the brain of 5XFAD AD mice, NRBF2 is found to interact with APP and recruit APP and APP- CTFs into autophagic structures and trigger their degradation in autolysosome. Besides, overexpression of NRBF2 decreases p62 but enhances LC3, which means that it is able to facilitates autophagy [98].

Transient receptor potential Mulcolipin-1 (TRPML1), which's expression is decreased in APP/PS1 transgenic mice, is involved in the initiation of autophagy by inhibition of mTOR and activation of AMPK signaling pathway. Overexpression of TRPML1 not only decreases the expression of Beclin1, LC3 and LAMP1, but further reduces cell viability and lysosomal ion concentration which have been impaired by Aβ_1-42_ [99].The triggering receptor expressed on myeloid cells 2 (TREM2) is an immune receptor which recruits PI3K through adaptor DAP 10 and promotes late-onset AD. Abundant LC3II and multivesicular structures with lower expression of p62 can be observed in 5XFAD with negative Trem2 expression, which shows that the deficiency of TREM2 further induces autophagy. And it is further identified that in the TREM2-deficient microglia from AD mice or human, mTOR is inhibited while AMPK is activated. Those reactions indicate that autophagy has been further induced and results in the removal of Aβ accumulated in microglia [100]. And it is also reported that deficiency of Toll-interacting protein (Tollip) disrupts endosome-lysosome fusion and promotes the accumulation of Aβ in neurons with an enhancement of p62, Parkin and the number of autophagosomes, which are involved in autophagy and mitophagy [101]. Therefore, the deficiency of TRPML1, TREM2 or Tollip in AD cases may have a positive effect to protect neurons via autophagy.

In addition, ErbB2 can physically dissociate Beclin-1 from the VPS34-VPS15 complex, and suppression of ErbB2 by an inhibitor promotes autophagy activation and decreases the level of βCTF and Aβ in AD models [102]. Hence, the presence of ErbB2is unbeneficial to the development of AD.

Recent studies have demonstrated that autophagy-related drugs or compounds, such as the mTOR inhibitor rapamycin, can rescue the cognitive deficits and remove the aggregates (such as Aβ and tau) in AD cases efficiently [74, 103]. Arctigenin, which is an extract from *Arctium lappa*, can induce autophagy by inhibiting AKT/mTOR pathway as well as activating AMPK/Raptor pathway, and then enhance Aβ clearance in cell and mouse models of AD [104]. The natural polyphenol resveratrol controls Aβ metabolism and mediates the anti-amyloidogenic effect through activating AMPK pathway, subsequently triggering the autophagic degradation of Aβ [105]. Functionalized single walled carbon nanotubes (SWNT) were found to restore normal autophagy by repairing aberrant activation of mTOR pathway and deficit in lysosomal proteolysis, which shows a novel neuroprotective approach in AD therapy [106]. Additionally, GTM-1 (a novel autophagy inducer) [107], latrepirdine (a pro-neurogenic, antihistaminic compound) [108], GSK-3 inhibitor (such as L803-mts) [109, 0], trehalose (a natural disaccharide) [78, 79], temsirolimus (a compound for renal cell carcinoma treatment) [111] and nilotinib (a drug for adult chronic myelogenous leukemia treatment) [112] also exert functions of autophagy induction and antagonism against Aβ toxicity in AD cases. Many active ingredients extracted from traditional Chinese herbs, like DDPU [113] (a ginsenoside derivative), berberine [114] (an isoquinoline alkaloid isolated from the coptidis Rhizoma), DNLA [115] (an active ingredientextracted from Dendrobium nobile Lindl) also have therapeutic effects in AD models. The autophagy targets of these compounds are listed in **Table 1**.

**Table 1.** Autophagy-related potential drugs for the treatment of neurodegenerative diseases.

**Disease**	**Drug**	**Autophagy targets**
AD	Rapamycin	mTORC1 inhibition
	Arctigenin	mTORC1 inhibition, AMPK activation
	Latrepirdine	mTORC1 inhibition
	Resveratrol	AMPK activation
	SWNT	mTORC1 inhibition, lysosomal proteolysis
	Nilotinib	PI3K CIII complex
	Trehalose	AMPK activation
	Temsirolimus	mTORC1 inhibition
	GSK-3β inhibitor, such as L803-mts	Lysosomal acidification
	GTM-1	Autophagosome maturation
	DDPU	mTORC1 inhibition
	Berberine	PI3K CIII complex
	Dendrobium nobile Lindl alkaloid (DNLA)	Autophagosome formation
PD	Curcumin	mTORC1 inhibition
	Resveratrol	AMPK activation
	Trehalose	PI3K CIII complex
	Lithium	mTORC1 inhibition
	Beclin-1 expression mimetics	PI3K CIII complex
	Nilotinib	Formation of autoplysosome
	Piperine	mTORC1 inhibition
	Sestrin2	AMPK activation
	Glycyrrhizic acid	PI3K CIII complex
	Calcitriol	AMPK activation
HD	Rapamycin,CCI-779	mTORC1 inhibition
	Rilmenidine	AMPK activation
	Acetylation at Lys444 of mutant HTT	HDAC modulation
	Histone deacetylase inhibitor	HDAC modulation
	Lithium	mTORC1 inhibition
	CTEP	Formation of autoplysosome
	Liraglutide	AMPK activation
	Neferine	AMPK activation
ALS	Rapamycin	TORC1 inhibition
	Lithium,VPA	Lysosomal acidification
	Trehalose	Autophagosome formation
	Berberine	mTORC1 inhibition
	S6K1 inhibitor	AMPK activation
	n-butylidenephthalide (BP)	mTORC1 inhibition

## PARKINSON'S DISEASE

Parkinson's disease (PD) is the second most prevalent neurodegenerative disease after AD, and it is characterized by selective loss of dopamine neurons in the substantia nigra (SN) and the presence of Lewy bodies, which are composed of α-synuclein and poly-ubiquitinated proteins [116]. In addition to motor syndromes such as resting tremor and muscular rigidity, PD patients also suffer from non-motor psychological and somatic symptoms [117], these influence human normal life seriously, and the main triggers of this disease are a combination of genetic predisposition and environmental factors.

In brains of PD patients, aberrant lysosomes and aggregated autophagosomes were observed in neurons [118], indicating a relationship between autophagy and PD. One of the pathological hallmarks of PD is accumulation of Lewy bodies, main components of which are misfolded and aggregated α-synuclein [119, 0]. The pathogenic role of autophagy in PD was revealed by increasing levels of α-synuclein when lysosomes are inhibited, and misfolded α-synuclein oligomers can be removed by different catabolic pathways including macroautophagy and chaperone-mediated autophagy (CMA) with different pathological situations, while α-synuclein monomers are also degraded by the proteasome [121, 122], suggesting a close link between α-synuclein degradation and autophagy. Furthermore, both over-expression of wild-type α-synuclein or A30P and A53T mutations of α-synuclein can inhibit autophagy [123, 124], and up-regulation of transcription factor EB (TFEB), a key autophagy modulator [125], could alleviate lysosomal damage by promoting its biogenesis, thus relieving α-synuclein associated pathology of neurodegenerative diseases [126, 127].

Emerging results have suggested that aberrant autophagy is one of the underlying mechanisms for PD, and this can be proved by evidences that several genetic mutations are linked to autophagy in hereditary forms of PD. In autosomal dominant PD, mutations in vacuolar protein sorting-associated protein 35 (VPS35) and leucine rich repeat kinase 2 (LRRK2) are mainly present. VPS35 is a retromer complex component, which recruits the actin nucleation-promoting WASP and Scar homolog (WASH) complex to endosomes. D620N mutation of VPS35 perturbs this recruitment and causes the mislocalization of mATG9 and defect of autophagosome formation [128]. LRRK2 exhibits pleiotropic functions, recent evidence raises the possibility that the toxic actions of LRRK2 are mediated by α-synuclein [129]. Shortened neurites and autophagosomes aggregation could be observed in differentiated SH-SY5Y cells expressing G2019S mutation of LRRK2 [130], which could cooperate with α-synuclein and cause age-related deficits of autophagy in a *C. elegans* model [131]. In the meantime, LRRK2 is able to recruit the PI3K III complex and Rubicon to the phagosome which inhibit the maturation of the phagosome [132]. Besides, mutations in SNCA (encoding α-synuclein), CHCHD2 (encoding a mitochondrial protein) and DNAJC13 (encoding a chaperon REM-8 involved in protein trafficking) are also related with autosomal dominant PD [117].

In autosomal recessive forms of PD, mutations in Parkin RBR E3 ubiquitin protein ligase (Parkin) [133] and PTEN induced putative kinase 1 (Pink1) [134] are the main pathogenic factors, accounting for 50% of familial cases in Europe [135]. A deficit in striatal synaptic plasticity and evoked dopamine release response was found in the striatum of mice where Parkin was deleted [136], and impaired activity of mitochondria was also observed in striatal neuron [137]. Similarly, deletion of Pink1 also leads to impaired respiration in striatal mitochondria and enhances sensitivity to oxidative stress in the cerebral cortex of mice [138]**.** Indeed, these two proteins act in the same way by selectively degrading damaged mitochondria to promote mitophagy [139, 140]. In this process, the proteasome-mediated degradation of Pink1 is stalled on damaged mitochondria, the accumulated Pink1 subsequently phosphorylates ubiquitin and recruits Parkin on the outer membrane of these mitochondria and results in their sequestration into autophagosomes. In the meantime, some outer membrane proteins are ubiquitinated by activated Parkin, and subsequently phosphorylated by Pink1, these linkages elicit a positive feedback involving more ubiquitinated proteins of mitochondria [139, 141–143]. Hence, a defect in mitophagy may be the cause in Pink1 or Parkin-positive familial forms of PD due to the accumulation of damaged mitochondria and excessive reactive oxygen species (ROS) production. In addition, the mutation of DJ-1 (a mitochondrial protein involved in the moderation of oxidative stress) is also related to this forms of PD, defective morphology and reduced activity are found in dopaminergic neurons derived from DJ-1 or Pink1 knockout mice [144]. In a rotenone induced PD rat model, the reduction of LAMP-2A protein, a marker of CMA, in dopamine neurons can be rescued by overexpression of DJ-1 in astrocytes, which indicates that astrocyte-specific DJ-1 overexpression has a positive effect on CMA [145]. And Fbw7βis a F-box protein which is involved in proteasomal degradation by interacting with Parkin and protects neurons from oxidative stress. A recent study has shown that 6-OHDA facilitates oxidation and the digestion of Fbw7β mainly by CMA. However, the level of Fbw7β did not change in postmortem PD brains compared to controls, thus needing further studies *in vivo* in PD patients [146].

Genome-wide association studies (GWAS) have identified a few lysosome related genes associated with PD. Mutations in the gene GBA (glucocerebrosidase β acid), encoding lysosomal hydrolase, disturb autophagosome-lysosome pathway and cause aggregation of α-synuclein [147, 148]. Lysosomal ATPases are enssential for the maintenance of lysosomal pH and, therefore, the activity of lysosomal proteases. The P-type ATPase ATP13A2 is found mutated in early-onset Parkinsonism [149, 150]. Mutations in ATP13A2 down-regulate degradation in lysosomes and accumulate α-synuclein protein in dopaminergic neurons [151, 152]. Recent studies show that depletion of ATP13A2 causes degradation of ubiquitinated synaptotagmin 11 (SYT11) that triggers lysosome dysfunction and impaired autophagosome degradation, and these can be rescued by overexpression of SYT11 in ATP13A2 knockdown cells [153]. Another ATPase, ATP6AP2, is required for lysosomal acidification and function, depletion of it has been related to X-linked parkinsonism with spasticity [147]. Moreover, VPS13C, having a function in maintaining the normal condition of lysosome and mitochondria, is involved in autosomal recessive Parkinsonism [147, 154], and the mutations of SCARB2, encoding lysosomal integral membrane protein-2 (LIMP-2), result in defects in autophagosome or lysosome function [155, 156]. Other abnormalities like oxidative stress also exhibit the involvement of autophagy in PD [157]. It has been shown that oxidative stress increases autophagic cell death in dopaminergic neurons by reducing the expression of Oxi-α, which encodes a novel mTOR activator [158]. TMEM175 is a component of the lysosome proteome which is important to regulate lysosomal pH and function. A study has discovered that higher levels of phosphorylated α-synuclein aggregates LC3 and p62 when TMEM175 is depleted in rat primary hippocampal neurons, which means a high risk of PD and damaged lysosomal degradation. In addition, TMEM175 is also involved in mitophagy via influencing mitochondrial respiration and regulating energy homeostasis. Thus, abnormal autophagy and mitophagy induced by TMEM175 deficiency might play an important role in the development of PD [159].

As representative candidate drugs for PD, resveratrol [160] and curcumin [161] have been reported to promote the degradation of α-synuclein by AMPK-SIRT1-autophagy pathway and mTOR/p70S6K signaling pathway respectively, both of them ameliorate the neurodegenerative pathology in cell models of PD. Trehalose enhances the clearance of mutant but not wild type α-synuclein in PC12 cells by activating autophagy [162], and nilotinib reverses motor behavior deficits and loss of dopamine neurons via autophagic degradation of α-synuclein in PD models [163]. A therapy of beclin 1 injections ameliorates the pathology of synapses and dendrites in PD model mice, and reduces α-synuclein aggregates, indicating that beclin-1 expression mimetics could be a kind of potential drugs for PD treatment [164]. Besides, piperine [165], sestrin2 [166], glycyrrhizic acid [167], calcitriolare [168] also exert anti-PD pathology properties in cell or mouse models, their specific autophagy targets are listed in **Table 1**.

## HUNTINGTON'S DISEASE

Huntington's disease (HD), an autosomal dominant neurodegenerative disease, is the most common polyglutamine disease. This kind of neurodegenerative disorder is caused by a CAG trinucleotide repeat expansion in the first exon of the huntingtin (HTT) gene which produces a mutant form of the HTT protein (mHTT) and leads to its pathogenic aggregation [169, 170]. Patients with HD suffer progressive motor, cognitive and psychiatric dysfunctions, which can be manifested by ataxia, chorea, dyskinesia, depression or memory and personality disturbances [117]. The pathogenic mechanism of HD is related to interferences in the key neuronal genes transcription, disturbances in the cytoskeletal system, impairments of mitochondrial activity and alterations in the autophagy-lysosome system [171].

There are aberrant relations between autophagy and the onset of HD. In the postmortem brains of HD patients, altered autophagy was observed [172], and activation of autophagy by rapamycin (a mTOR inhibitor) treatment shows a neuroprotective effect and attenuates HTT toxicity in a fly model of HD [173]. Moreover, an altered expression of autophagy-related genes has been discovered in HD patients. In this aspect, the expression of genes such as LC3I, ULK2 and LAMP2 are increased in mRNA level, while the expression of EEF1A2, FKBP1A and PINK1 is down regulated [174]. A recent study showed that the V471A polymorphism in ATG7 is related to an earlier onset form of HD [175]. The exact mechanism of autophagic dysfunction in HD is poorly understood, but the inefficient degradation of autophagosomes may be the cause of their slower turnover and HTT accumulation in HD cells. This can be proved by aggregated autophagosomes observed in cellular and animal models of HD, thus dysfunction of loading into autophagosomes causes an impaired autophagic protein degradation [176].

Wild type HTT plays a key role in axonal transport of autophagosomes together with Huntington associated protein 1 (HAP1) [174]. Depletion of HTT in HD models results in abnormal accumulation of autophagosomes [177], and HTT also shares resemble structure with ATG11 and mTOR to join in the formation of autophagosome [56, 178]. Together with the observation that overexpression of full-length HTT stimulates activation of autophagy and promotes clearance of its mutant form [179], it is tempting to speculate that wild type HTT may have extensive interactions with autophagic pathways in HD. Further studies revealed that HTT interacts with autophagy-associated proteins to influence autophagy pathway indirectly. HTT reduces the activity of mTOR by competing with mTOR in binding to ULK1, as a result, initiation of autophagy is evoked [180, 181]. Additionally, it also acts as a scaffold [182] to support translocation and binding of p62, ubiquitinated proteins and LC3 to enhance autophagy activation [180]. It's reasonable to suppose that wild type HTT may regulate autophagy in different ways and that dysfunctional autophagy may also be implicated in HD cases.

It seems that heterozygous forms of HD are more common in the present studies, which means that patients carry a functional HTT along with mHTT [183]. mHTT displays different properties and functions compared to normal HTT due to its expanded region of glutamine residues, and the interaction between mHTT and its target proteins can be determined by the length of its poly-glutamine (polyQ) tract [183, 184]. Compared with wild type HTT, mHTT seems to mediate autophagy by different ways. In addition to pathogenic aggregation of mHTT which aggravates the condition in HD, soluble forms of mHTT also represent cytotoxicity by interacting with regulators of autophagy like beclin-1, and both of the forms can be degraded by autophagy [185, 186].

The normal functions of HTT are essential for neuronal development. Studies found that autophagosome transport is inhibited by either loss of HTT or expression of the mutant protein in striatal neurons, subsequently obstructing the fusion of autophagosome and autolysosome [187]. Further studies found that mHTT can also interact with p62 instead of wild type HTT, leading to dysfunction of p62 to recognize cargo aggregates and organelles, causing abnormal autophagy and proteasome degradation [188, 189]. Besides, mHTT is able to trigger chronic stress and prolonged unfolded protein response (UPR), resulting in lower aggregate removal and inhibition of autophagy via IRE1 [190]. Recent experiments revealed that mHTT has the ability to compete with Ataxin-3 to capture Beclin 1 via its polyQ, leading to impairment of starvation-induced autophagy in neurons [184, 191]. In other words, Beclin 1 can be recruited by mHTT directly, which may be a reason for unsuccessful Beclin 1-mediated long-lived protein turnover and reduction of mHTT degradation in HD cases [184, 185].

Rhes, which is required for autophagy by interacting with Beclin 1 and facilitating disassociation between Bcl-2 and Beclin 1 [192], is invalid when interacting with mHTT which causes the impairment of autophagy initiation [192]. mTOR, another negative regulator of autophagy, is separated by mHTT which forms aggregates around mTOR, thus reducing its activity in HD and SCA7 brains [173, 189, 193].

Moreover, the activity of some enzymes also plays a role in regulating the effect of autophagy to remove mHTT aggregates. For example, up-regulation of casein kinase 2 (CK2) reduces large inclusion formation of mHTT by phosphorylating p62[194]. Down-regulation of Phosphatidylinositol-5-phosphate 4-kinase, type II γ (PIP4Kγ) enhances basal autophagy and reduces the aggregates and total amount of mHTT protein in neurons and fibroblasts respectively and rescues mHTT-induced neurodegeneration in two *Drosophila* HD models [195]. In mHTT-expressing neuro2A, Glycogen synthase (GS) is activated and promotes autophagy and this response is specific in neurons. Co expression of GS and mHTT can be found in HD-associated cells which restores autophagy whereas excessive autophagy is easy to cause neuronal death [196]. Overall, mHTT has multidirectional effects on the regulation of autophagy, the ratio of soluble to aggregated mutant protein may determine the toxic or protective outcome [197].

It is worth to mention that the classic inhibitor of mTOR, rapamycin or its analogue CCI-779, can alleviate severity of Huntington-like phenotype in behavioral experiments and facilitate the clearance of mHTT aggregates in a mouse model of HD [173], and co-treatment of rapamycin and trehalose in mice has a synergistic effect on the induction of autophagy which may accelerate the degradation of these aggregate-prone proteins efficiently [198]. Besides, lithium may be a potential drug for the treatment of PD and HD, for it has ability to remove the abnormal accumulations of mHTT and α-synuclein by inhibiting inositol monophosphatase and thus inducing autophagy [199]. Moreover, acetylation at Lys444 of mHTT [200] and upregulation of HSC70 and lamp2A [201] have been regarded as the novel therapies to remove mHTT by autophagy. Additionally, some drugs may also be useful for the treatment of HD, such as rilmenidine [202], histone deacetylase (HDAC) inhibitors [203], CTEP (a negative allosteric modulator of metabotropic glutamate receptor 5 (mGluR5)) [204], liraglutide (a GLP-1 analogue) [205], neferine (a bisbenzylisoquinoline alkaloid isolated from the lotus seed embryo of *Nelumbo nucifera* Gaertn) [206], their targets are listed in **Table 1**.

## AMYOTROPHIC LATERAL SCLEROSIS

Amyotrophic lateral sclerosis (ALS) is a fatal neurodegenerative disease with the symptoms of muscle weakness, spasticity and atrophy. Selective loss of motor neurons can be observed in the brain and spinal cord of the patients [207, 208]. Environmental elements such as exposure to toxic substances or heavy metals raise the risk for developing ALS. The genetic mutations such as superoxide dismutase 1 (SOD1), TAR DNA-binding protein 43 (TDP-43) and Chromosome 9 open reading frame 72 (C9ORF72), fused in sarcoma/translocated in lip sarcoma (FUS/TLS) resulting in accumulation of misfolded proteins have been linked to the disease [209]. Like other neurodegenerative diseases, there are sporadic and familial forms of ALS, the sporadic form is seen in the majority of known cases, while the family ones account for approximately 5%-10% [210].

Numerous studies try to define the molecular pathogenesis of these devastating diseases, recently it has become apparent that the autophagic/lysosomal system dysfunction is tightly associated with ALS. Indeed, aggregated autophagosomes in the cytoplasm observed in the spinal cord of sporadic ALS patients indicate that autophagy is activated [211]. Immunohistochemical analysis in a mutant SOD1 ALS mouse model (SOD1G93A) has shown the activation of autophagy [212]. Increased autophagosome formation and decreased phosphorylation of mTOR/Ser2448 are also found in motor neurons of SOD1G93A transgenic mice, indicating that autophagy dysfunction possibly underlies pathological phenomena in ALS [213].

SOD1 is the most common mutated gene in ALS, and the toxic gain-of-function mutations in this gene lead to its misfolding and aggregation [214]. The two studies in SOD1 mutant mice mentioned above have shown that mutant SOD1 enhanced the function of mTOR-dependent autophagy [212, 213]. Besides, p62 is also increasing at the same time, which shows that autophagy fails to degrade cellular products caused by SOD1 mutations [215, 216]. Furthermore, knockdown of the UPR transcription factor X-box-binding protein-1(XBP-1) in mice stimulates autophagy and promotes digestion of mutant SOD1, which can hinder the development of ALS [217]. And the heat-shock protein (HspB8) enhanced the ability to remove aggregation and mutant SOD1 by promoting autophagy in an ALS model [218]. The efficient autophagy clearance of mutant SOD1 may be beneficial for reducing motor neuron loss in ALS.

Many reports have revealed that a number of autophagy receptors are encoded by ALS-linked genes, p62/SQSTM1 is one of them. p62 contains SMIC, UBA and LIR domains, which can bind to SOD1, TDP-43, and LC3, respectively [219, 220]. LC3 fails to recognize p62 when ALS-associated L341V mutation occurs in such cells, whereas ubiquitinated proteins still bind to it, thus causing mutant p62 and its binding protein unable to be recruited into phagophores and interrupting the autophagy-mediated degradation pathways [219–221].Consequently, this leads to the accumulation of mutant SOD1 and TDP-43, further accelerating the development of ALS. Furthermore, over-expression of p62 could relieve TDP-43 aggregation by autophagy or proteasome pathway *in vitro* [222].

Another autophagy receptor which is associated with ALS is optineurin (OPTN). OPTN is a ubiquitin-binding scaffold protein and take part in selective autophagy processes. Its activity is regulated by TANK-binding kinase 1 (TBK1), a protein involved in autophagy by phosphorylating p62, OPTN [223]. Inhibiting the expression of TBK1 interrupts efficient formation and maturation of autophagosomes [224, 225]. Besides, OPTN also interacts with myosin VI, and autophagosome-lysosome fusion will decrease by ALS-associated mutations in the myosin VI-binding domain of OPTN, indicating OPTN is required for autophagosome trafficking [226, 227]. Furthermore, OPTN and TBK1 influence mitophagy. Pink1 and Parkin can recruit TBK1 and OPTN which act as autophagic receptor to mitochondria membrane, so that they accelerate recruitment of LC3 and promote digestion of damaged mitochondria. ALS-associated mutation in OPTN and TBK1 block the closure of depolarized mitochondria and induce the accumulation of damaged mitochondria, which can break cell homeostasis, especially in neurons [225, 228, 229].

Ubiquilin2 (UBQLN2), which acts as a proteasome shuttle factor, has an ability to mark the protein with ubiquitin label for autophagy degradation, therefore it plays a crucial role in formation of autophagosome [230]. Mutations in UBQLN2 cause dominantly inherited ALS, resulting in neuron loss, cognitive deficits and shortened lifespan in mouse models [231, 231]. Mutant UBQLN2 combines with polyubiquitinated proteins prior to proteasome, leading to a defect in proteasomal degradation and accumulation of misfolded proteins [233, 234].

C9ORF72 is the most common genetic factor giving rise to ALS, and mutations in the hexanucleotide-repeat expansion of C9ORF72 gene cause disease through a number of different mechanisms [235, 236]. C9ORF72 is reduced in ALS and FTD. When C9ORF72 is deleted in neurons, the accumulation of aggregated p62 and TDP-43 will occur in the cell apparently [237]. Meanwhile, decreased activity of mTOR accompanied by enlarged lysosomal compartments and enhanced autophagic flux were found in C9ORF72 depletion cells, suggesting that C9ORF72 is related to mTOR-dependent autophagy [238]. In addition, C9ORF72 forms a complex with WDR41 and SMCR8 [239, 240]. This complex acts as a GDP/GTP exchange factor (GEF) to activate Rab8a and Rab39b, thus affecting the formation or maturation of autophagosomes [224, 241]. In addition, this C9ORF72 complex interacts with the ULK1 complex and is required for translocation of the later one. Loss of SMCR8 leads to a similar phenotype as C9ORF72 depletion and results in defective autophagy, indicating that this interaction is required for modulating autophagy induction [242].Whereas a recent study shows a new topic that depletion of C9ORF72 is not deleterious by itself but synergizes with Ataxin-2 toxicity to impair motor neuron's function and lead to neuronal cell death, thus revealing a double-hit pathological mechanism in ALS [237].

An additional familial ALS gene has been reported, namely endosomal sorting complexes required for transport (ESCRT). It is required to form functional multi-vesicular bodies (MVBs) and mediates its internalization process so that most of the substances can be degraded by autophagy. ESCRT and its subunit charged multi-vesicular body protein-2B (CHMP2B) have been identified to be associated with ALS. Depletion of ESCRT or mutation in CHMP2B inhibited autophagy due to impaired autophagosome-lysosome fusion, resulting in the accumulation of ubiquitin-positive proteins and p62 [42, 243]. Besides, dysfunctional MVBs in ESCRT mutated cells weaken the ability to remove TDP-43, which is the main misfolded proteins in ALS, also ensuring the connection between ESCRT and ALS [244, 245].

Recent studies have shown that progranulin (PGRN), a secreted growth factor, and Sigma receptor-1 (SigR1), an ER chaperone, contribute to the pathogenesis of ALS and both of them own functions in neuronal survival [246, 247]. The deficiency of PGRN promotes the formation of TDP-43 aggregates and inhibits autophagy in neurons [246]. The ALS-linked E102Q mutant SigR1 aggregates, co-localizes with TDP-43 in the inclusion, leading to accumulation of p62 and LC3II and obstructing autophagosome-autolysosome fusion [247].

Mutant Valosin-containing protein (VCP) also has been discovered in ALS patients, and it seems to regulate autophagosome removal [248]**.** VCP is an indispensable component to maintain the integrity and dynamics of the lysosomal network and is subsequently implicated in the maturation and fusion of autophagosome and autolysosome [248–250]. A study found that mutant VCP in ALS interacts with TDP-43 genetically and causes the redistribution of TDP-43 to the cytoplasm, thus probably acting as a etiology of ALS [251, 252].

It is corroborated that rapamycin also exerts a positive effect on the therapy of ALS by a mTOR-dependent pathway, however, its function has been argued that it cannot remove the aggregates apparently in mice expressing abnormal SOD1 [253, 254]. The treatment with lithium is able to alleviate the symptoms of ALS in human and animal cases by triggering autophagy through the GSK-3β pathway, and the collaboration of lithium and valproic acid (VPA) may have a better therapeutic effect on ALS [255, 256]. Trehalose can upregulate the expression of ATG5, LC3 and beclin1, and subsequently promote the formation of autphagosome to delay disease onset and prolong lifespan [257]. Besides, berberine [258], p70 S6 kinase 1 (S6K1) inhibitors [259] or n-butylidenephthalide (BP) [260] are also involved in the autophagy related therapy of ALS, their targets are listed in **Table 1**.

## CONCLUSIONS

**Figure 2:**
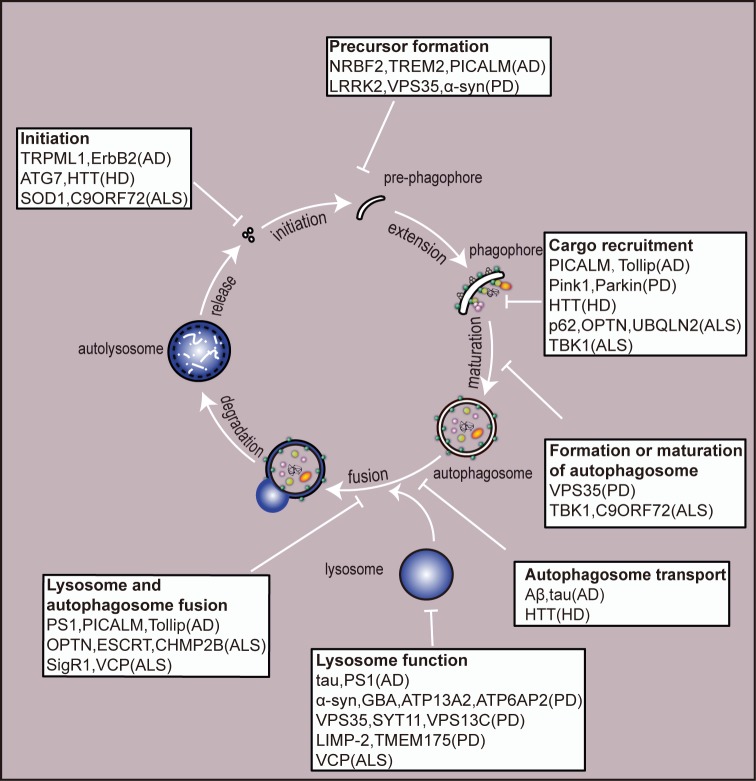
FIGURE 2: An overview of the autophagy pathway and the site of action of disease-associated proteins. A huge number of neurodegenerative disease-related genes have been implicated in autophagy function. Mutation or deletion of these genes have been suggested to be involved in perturbation throughout the autophagic process, from initiation of autophagosome formation to degradation in the autolysosomes. Their proposed sites of action are highlighted in boxes. Please note that some disease-associated proteins act at multiple points in the process. AD - Alzheimer disease; PD - Parkinson's disease; HD - Huntington's disease; ALS - amyotrophic lateral sclerosis.

Autophagy acts as a ubiquitous degradative pathway of large protein aggregates and damaged organelles to maintain homeostasis and function of the neurons. To date, numerous studies have shown that autophagy plays an important role in the onset and development of neurodegeneration. To sum up, abnormal proteins which give rise to neurodegenerative diseases, such as Aβ in AD, α-synuclein in PD, HTT in HD and SOD1 in ALS, will modulate autophagy in a different manner. Dysfunction in the process of autophagy pathway, such as vesicular transportation, autophagosome formation and autophagosome-autolysosome fusion, may cause the accumulation of abnormal proteins in neurons which may exacerbate the damage of neurons. Hence, the dysfunction of autophagy process is implicated in the pathology of neurodegenerative diseases. A detailed illustration of autophagic alternations in neurodegenerative diseases is shown in **Figure 2**.

It seems that the proper enhancement of autophagy may be beneficial for cell survival in neurons. Thus, autophagy will become a therapeutic target to ameliorate neurodegenerative diseases. However, the mechanism of autophagy in neurodegenerative diseases and the crosstalk between autophagy and other regulatory system, such as the immune process and inflammation, is complicated and unclear. Moreover, the specific therapeutic target of autophagy and the signal pathways involved is also undiscovered. There are still many unsolved mysteries that need further exploration.

## Abbreviations:

Aβ: – amyloid beta,
AD: – Alzheimer's disease,
ALS: – amyotrophic lateral sclerosis,
AMPK: – AMP-activated protein kinase,
APP: – amyloid precursor protein,
ATG: – autophagy related,
AV: – autophagic vacuole,
C9ORF72: – Chromosome 9 open reading frame 72,
CMA: – chaperone-mediated autophagy,
CTF: – C-terminal fragment,
ESCRT: – endosomal sorting complexes required for transport,
FTD: – frontotemporal dementia,
HD: – Huntington's disease,
HTT: – huntingtin,
mHTT: – mutant HTT,
mTOR: – mammalian target of rapamycin,
MVB: – multi-vesicular body,
PD: – Parkinson's disease,
PI3K: – phosphatidyl inositol 3-kinase,
polyQ: – poly-glutamine,
VCP: – valosin-containing protein.
